# Factors Associated to Duration of Hepatitis A Outbreaks: Implications for Control

**DOI:** 10.1371/journal.pone.0031339

**Published:** 2012-02-15

**Authors:** Nuria Torner, Sonia Broner, Ana Martinez, Cecilia Tortajada, Patricia Garcia de Olalla, Irene Barrabeig, MariaRosa Sala, Neus Camps, Sofia Minguell, Josep Alvarez, Gloria Ferrús, Roser Torra, Pere Godoy, Angela Dominguez, the Hepatitis A Surveillance Group of Catalonia, Spain

**Affiliations:** 1 Department of Health. Generalitat of Catalonia. Barcelona, Spain; 2 CIBER Epidemiologia y Salud Pública (CIBERESP), Barcelona, Spain; 3 Department of Public Health. University of Barcelona, Barcelona, Spain; 4 Public Health Agency of Barcelona, Barcelona, Spain; Saint Louis University, United States of America

## Abstract

Even though hepatitis A mass vaccination effectiveness is high, outbreaks continue to occur. The aim of this study was to investigate the association between duration and characteristics of hepatitis A outbreaks. Hepatitis A (HA) outbreaks reported between 1991 and 2007 were studied. An outbreak was defined as ≥2 epidemiologically-linked cases with ≥1 case laboratory-confirmed by detection of HA immunoglobulin M (IgM) antibodies. Relationships between explanatory variables and outbreak duration were assessed by logistic regression. During the study period, 268 outbreaks (rate 2.45 per million persons-year) and 1396 cases (rate 1.28 per 10^5^ persons-year) were reported. Factors associated with shorter duration were time to intervention (OR = 0.96; 95% CI: 0.94–0.98) and school setting (OR = 0.39; 95% CI: 0.16–0.92). In person-to-person transmission outbreaks only time to intervention was associated with shorter outbreak duration (OR = 0.96; 95% CI: 0.95–0.98). The only variables associated with shorter outbreak duration were early administration of IG or vaccine and a school setting. Timely reporting HA outbreaks was associated with outbreak duration. Making confirmed HA infections statutory reportable for clinical laboratories could diminish outbreak duration.

## Introduction

Hepatitis A infection takes place mainly when a non-immune individual consumes contaminated food or water or is in contact with feces of a person in the phase of shedding the virus. In adults the clinical manifestations of hepatitis A are indistinguishable from those of other viral hepatitis including discomfort, anorexia and jaundice lasting between two weeks and several months. Although transmission to the fetus is unusual, there are some case reports in which mothers developed hepatitis A during the first trimester of pregnancy and their infants developed meconium peritonitis. In children hepatitis A virus infection (HAV) is usually asymptomatic but the virus is shed in the feces in asymptomatic infections and therefore infected children are an important source of infection [1].

Hepatitis A and B vaccinations integrated into public health procedures and universal immunization programs are the best way to accomplish elimination of VHA infection in most settings. Although hepatitis vaccines should be given to all susceptible persons at risk, many opportunities to vaccinate adults at high risk are missed [2]. At the end of 1998, a mass vaccination program with a combined hepatitis A+B vaccine was initiated in 12-year-old preadolescents in Catalonia. Seven years after the introduction of universal hepatitis A vaccination in Catalonia, the incidence rate declined more than 45%. The fact that the greatest reduction (72.3%) occurred in the 10–19 years age group, which included the cohorts vaccinated after 1998 suggested that vaccination played an important role in the decline. Nevertheless, even though the effectiveness of mass vaccination of preadolescents has been estimated at >99% [3], [4],outbreaks continue to occur in Catalonia, as in other countries with mass vaccination programs [5], [6].Outbreak investigation imply reporting suspected cases to the public health services and interventions to prevent exposure of contacts. Outbreak duration is an important public health issue because the resources involved increasing with the period of time that these resources are focused to control activities.

The objective of this study was to investigate the association between the characteristics of hepatitis A outbreaks reported in Catalonia and their duration.

## Methods

The study was carried out in Catalonia, a region with seven million inhabitants situated in the northeast of Spain. Hepatitis A clinical case was defined as an acute illness with discrete onset of symptoms (malaise, nausea, anorexia, fever, malaise, or abdominal pain) and jaundice, dark urine or elevated serum aminotransferase levels. A confirmed case was considered as one that meets the clinical case definition and is laboratory confirmed by Immunoglobulin M (IgM) antibody to hepatitis A virus (anti-HAV) detection or a case that meets the clinical case definition and occurs in a person who has an epidemiologic link with a person who has laboratory-confirmed hepatitis A during the 15–50 days before the onset of symptoms. A hepatitis A outbreak was defined as ≥2 epidemiologically-linked cases with at least one case laboratory-confirmed by detection of anti-HAV IgM. Physicians must notify outbreaks to the units of epidemiological surveillance urgently (before 24 hours of suspicion). All hepatitis A outbreaks reported from January 1991 to December 2007 were studied.

Data collected included the number of cases, median age, setting of the outbreak and whether the cases were immigrants. The dates of onset of symptoms of the first and last cases were used to determine outbreak duration. Interventions such as the administration of standard human immunoglobulin (IG) or vaccine and the date of administration were also recorded. This date and the date of onset of symptoms of the first case were used to determine the time to intervention. The delay in reporting the outbreak was assessed by comparing the date of onset of the first case and the date of reporting to the surveillance unit. The analysis was carried out considering all reported outbreaks and, separately, person-to-person outbreaks. The dependent variable was duration of the outbreak above and below 50 days, which is the maximum number of days of the incubation period of the disease. It some covariables were kept as continuous (time to intervention and reporting delay) because they fitted the linear assumption that the logit is linear in the variable. Other continuous variables (size and age) that didn't meet the linear assumption were considered as categorical according to whether values were equal to or below the median or above the median. Binary variables included were school setting/other setting, cases in immigrants/cases in others, and administration of IG or vaccination/no administration. Co-linearity between independent continuous variables was assessed by Spearman correlation tests, before they were transformed to binary. A logistic regression model was used to assess the relationship between explanatory variables and outbreak duration. Explanatory variables were first assess by univariate analysis and adjusted with a multivariable analysis. Final model adjustment was determined by the Hosmer and Lemeshow test [7]. Statistical significance was set at alpha error = 0.05. The analysis was carried out using the Statistical Package of Social Sciences (SPSS 19.0 for Windows). No ethics consent was required for the study which is based on anonymous information extracted from outbreak investigation reports.

Explanatory variables were first assess by univariate analysis and adjusted with a multivariate analysis. Final model adjustment was determined by the Hosmer and Lemeshow test [7]. Statistical significance was set at alpha error = 0.05. The analysis was carried out using the Statistical Package of Social Sciences (SPSS 19.0 for Windows). No ethics consent was required for the study which is based on anonymous information extracted from outbreak investigation reports.

## Results

During the study period, 268 outbreaks (rate 2.45 per million persons-year) were reported that involved 1396 patients (rate 1.28 per 10^5^ persons-year) and information about intervention measures was known in 118 outbreaks (44%), involving 812 cases. Considering the 118 outbreaks for which information was available, 97 (82.2%) had person-to-person transmission, 17 (14.4%) a common source and in 4 outbreaks (3.4%) the transmission was unknown. The most frequent setting for all outbreaks studied was the school (49.2%). In person-to-person outbreaks, 53.6% of outbreaks occurred in schools or day-care centres.

Colinearity analysis found correlation between the independent explanatory variables size, time to intervention and delay. Therefore, models were specified considering these as separate variables.

The results of the univariate and multivariable logistic regression analysis are shown in tables 1 and 2. The factors associated with a shorter duration in the univariate analysis were: size of the outbreak ≤4 cases (OR = 5.91; 95% CI: 2.64–13.23), time to intervention (days) (OR = 0.96; 95% CI: 0.94–0.98), reporting delay (days) (OR = 0.96; 95% CI: 0.94–0.99) and school setting (OR = 0.40; 95% CI: 0.19–0.85). The variables age, immigrant status and administration of IG or vaccine were not associated to duration. The multivariable analysis for one of the fitted models showed a statistically significant negative association between time to intervention (OR = 0.96; 95% CI: 0.94–0.98) and also a negative association with school setting (OR = 0.39; 95% CI: 0.16–0.74). In person-to-person transmission outbreaks, the variables associated with a shorter outbreak duration in the univariate analysis were also outbreak size (OR = 6.59; 95% CI: 2.71–16.07), time to intervention (OR = 0.96; 95%CI: 0.95–0.98) and reporting delay (OR = 0.96; 95%CI: 0.94–0.98); while in the multivariable analysis only time to intervention was associated with a shorter outbreak duration (OR = 0.96; 95%CI: 0.94–0.98).

**Table 1:** Association between outbreak duration and independent variables in the multivariable analysis: all outbreaks and person-to-person outbreaks.

	OR crude(95%CI)	p value	OR adjusted(95%CI)	P value
Size				
>4 cases	1			
≤4 cases	5.91 (2.64–13.23)	<0.001		
Time to intervention (days)	0.96 (0.94–0.98)	<0.001	0.96 (0.94–0.98)	<0.001
Administration of IG or vaccine				
No	1			
Yes	0.49 (0.22–1.11)	0.087		
School setting				
No	1		1	
Yes	0.40 (0.19–0.85)	0.017	0.39 (0.16–0.92)	0.031
Immigrant status				
No	1			
Yes	1.91 (0.68–5.34)	0.218		
Delay in reporting (days)	0.96 (0.94–0.99)	<0.001		
Age				
>10 years	1			
≤10 years	1.16 (0.55–2.42)	0.700		

**Table 2:** Association between outbreak duration and independent variables in the multivariable analysis of person-to-person outbreaks.

	OR crude(95%CI)	p value	OR adjusted(95%CI)	P value
Size				
>4 cases	1			
≤4 cases	6.59 (2.71–16.07)	<0.001		
Time to intervention (days)	0.96 (0.95–0.98)	<0.001	0.96 (0.95–0.98)	<0.001
Administration of IG or vaccine				
No	1			
Yes	0.47 (0.20–1.19)	0.088		
School setting				
No	1			
Yes	0.47 (0.21–1.07)	0.073		
Immigrant status				
No	1			
Yes	2.27 (0.79–6.53)	0.127		
Delay in reporting (days)	0.96 (0.94–0.98)	<0.001		
Age				
>8.8 years	1			
≤8.8 years	1.40 (0.63–3.13)	0.413		

The model that takes into account size and the remaining non-correlated variables, size is the only variable presenting statistical significance. Whereas the model considering delay, both delay and school setting were statistically significant, thus the latter seems the model that best fits to highlight how the variable time to intervention influences duration of outbreaks.

## Discussion

The majority of outbreaks in this study were due to person-to-person transmission and the most frequent setting was the school. Only a small proportion (14.4%) of outbreaks included in this study were foodborne. Common source hepatitis A outbreaks are difficult to detect due to the high proportion of asymptomatic cases and the long incubation period that makes recall of exposure difficult, nevertheless the importance of person-to-person transmission is undeniable in our environment [8]. Another fact that adds impairment to detection being that, because hepatitis A is highly endemic in many developing countries, those temporarily returning to their countries of origin fail to consult health professionals prior to departure and thus render vaccination programs for travellers ineffective [9]–[11].

The duration of hepatitis A outbreaks is important in public health because a lot of resources are invested in characterizing new cases and determining which control measures are needed. In addition to personal hygiene and environmental measures, post-exposure prophylaxis includes administration of IG and the administration of one dose of vaccine to susceptible contacts [12]. Both IG and the vaccine should be administered within two weeks after exposure in order to obtain the desired shortening of outbreak duration. However, the cost per case in outbreaks where IG was administered was estimated as up to 12,766 US dollars in a school outbreak and 5,545 US dollars in a defined risk group of adults, with the cost of the intervention representing a quarter of the total costs [13]. In addition to logistic difficulties, although IG can prevent clinical disease, some individuals continue to have subclinical infections that may contribute to transmission [14]. The efficacy of vaccine administration as post-exposure prophylaxis has been demonstrated in a well-designed randomized double-blind trial [15]. Because the use of vaccine as a post –exposure prophylaxis is more recent, there are less evaluation studies but the cost of this intervention in school setting seems lower [16].

Universal hepatitis A vaccination programs may result in great benefits. The introduction of universal vaccination of children has represented a saving of 3,660 doses of IG in Israel [17] when hepatitis A outbreaks avoided were considered. In addition, universal vaccination provides strong herd immunity capable of interrupting chains of transmission in the general population. But in the post-vaccination era, hepatitis A outbreaks can still occur, as shown in this study and to improve the knowledge about which factors are determinant for their control is important. In the present study, the only variables associated with a shorter duration of any outbreak (including common-source outbreaks) was early administration of IG or vaccine and school setting. This result is in accordance with other reports of effective control of the outbreaks if the intervention is administered promptly. Shen et al [18] demonstrated that the effectiveness of post-exposure vaccination was 100% in a common-source outbreak, but Hauri et al found post-exposure vaccination did not control a person-to-person outbreak because the intervention was not rapid enough [19].

Assuming that there is under-detection due to some asymptomatic infections, which are equally infectious for susceptible people, the detection of all confirmed infections in addition to clinical cases could improve outbreak detection.

Because an outbreak can be reported by a physician only when two or more suspected cases are epidemiologically linked and frequently this is only possible if more cases occur, a probable way of improving outbreak detection is by making confirmed HAV infections (with or without symptoms) a statutorily reportable situation for clinical laboratories. This would make possible earlier epidemiological A limitation of this study to be taken into consideration, is that standard outbreak investigation reports do not include as mandatory data information about the timing of control interventions. Consequently this variable was available in only 44% of reported outbreaks, which in turn were also the largest ones.The mean number of cases was 5.2 for all reported outbreaks during the study period, compared with 6.9 for outbreaks included in the study. However, as larger outbreaks consume most public health resources, our results may be of interest for control purposes.

In summary, this study shows that timely intervention is the most important factor associated with outbreak duration. The link between surveillance units and laboratories has been shown to be positive in foodborne disease outbreaks allowing the implementation of public health interventions to prevent further cases [20]. Thus modifications of regulatory norms for reporting hepatitis A virus infection in clinical laboratories should be introduced to diminish outbreak duration.
